# Barriers to Implementing On-Site Companion Animal Programs in U.S. Domestic Violence Shelters: Does Shelter Location Matter?

**DOI:** 10.1177/08862605251351668

**Published:** 2025-07-10

**Authors:** Patti Timmons Fritz, Claire Baldwin, Allison Gray, Betty Barrett, Amy Fitzgerald, Rochelle Stevenson

**Affiliations:** 1 University of Windsor, Windsor, ON, Canada; 2 Welcome Centre Shelter for Women and Families, Windsor, ON, Canada; 3 Western University, London, ON, Canada; 4 Thompson Rivers University, Kamloops, BC, Canada

**Keywords:** intimate partner violence, animal abuse, pet programs, the link, obstacles

## Abstract

Women in abusive relationships who are not able to take their companion animals (CAs) with them to domestic violence shelters report staying with their abusive partners longer. Many domestic violence shelters are therefore considering establishing CA programs to address this concern. However, little research has examined existing on-site CA programs, or the barriers shelters face in establishing them. The purpose of the current study was to investigate the barriers domestic violence shelter staff face in developing and implementing on-site CA programs. Contact was attempted with 1,740 domestic violence shelters across the United States, 702 shelters (40.3%) completed the survey through telephone interviews and online surveys, and 405 indicated that they did not have an existing on-site CA program in place. Results showed that health and safety (43.6%), space (40.6%), and resources (13.1%) were the most frequently reported barriers, that most shelters identified only one or two barriers, and that the nature of the primary barriers as well as the number of barriers endorsed did not significantly differ across rural versus urban locations or geographical regions of the United States (*p*s > .05). Findings suggest there is a need for developing strategies for implementing on-site CA programs, and that these strategies can be used across the United States to help intimate partner violence survivors and their CAs seek safety.

Research on intimate partner violence (IPV) and its link to companion animal (CA) maltreatment is a growing field of study. IPV is “a pattern of behavior that is used to gain or maintain power and control over an intimate partner through the use of physical, sexual, emotional, economic, or psychological actions or threats of action that influence the victim” (United Nations, 2021). Although some research has examined the relations between IPV and animal maltreatment (aka “the link”), less is known about the barriers shelters face in establishing on-site CA programs to help IPV survivors fleeing with CAs. Moreover, there is a need to explore whether shelters residing in different locations and geographical regions experience different barriers (e.g., to inform decisions about resource allocation). The current study explored these gaps in the literature.

## IPV and Animal Maltreatment

The co-occurrence of IPV and animal maltreatment is a serious social and public health issue. Between 25% and 89% of women in domestic violence shelters in North America report that their abusive partners have also mistreated or threatened to mistreat their CAs (Ascione, 1998; Barrett et al., 2020; Carlisle-Frank et al., 2004; Collins et al., 2018; Faver & Cavazos, 2007; Faver & Strand, 2003; Flynn, 2000; Simmons & Lehmann, 2007; Strand & Faver, 2005). When considering severity, Barrett et al. (2020) found that of 55 women in domestic violence shelters who had a CA when involved with an abusive partner, 62% reported that their CA suffered high or severe levels of abuse whereas 38% reported that their CA was either not abused or the animal experienced minor levels of abuse. Results of this study showed that 56% of women surveyed delayed leaving their partner out of concern for the safety of their CA, and that 60% left their CAs with their abuser to flee to a domestic violence shelter. Other research has similarly documented IPV victims’ delayed leaving due to fears for their CAs’ safety and well-being, as well as the intense emotional turmoil victim-survivors—and their children—experience when they must leave their CAs behind (Giesbrecht, 2022a, 2022b; Giesbrecht et al., 2025). These data raise safety concerns not only for victims staying out of concern for their CA but also for the CAs left behind with abusive partners (Barrett et al., 2020; Giesbrecht, 2022b).

One factor contributing to the difficulty in leaving a CA behind when fleeing from IPV may be the bond shared between a victim-survivor and the CA. Surveys show that the vast majority of those who share their homes with CAs view their CAs as members of the family (Ingraham, 2019). Many report the human–CA bond to be close to the one shared between a parent and a child (Castillo, 2013; Giesbrecht, 2022a). The support an individual can receive from a CA can further the bond, thus making it that much more difficult for an IPV victim to leave without the CA. Given abusers’ tendencies to isolate their victims from family and friends, CAs may be the only social support some victims have, thus making the CA essential to the victim (Barrett et al., 2018). The bond between an individual and their CA can also be extorted by perpetrators of IPV (Ascione et al., 2007; Barrett et al., 2020; Collins et al., 2018; Fitzgerald et al., 2019; Giesbrecht, 2022a, 2022b; Hardesty et al., 2013; McDonald et al., 2015; Simmons & Lehmann, 2007; Wuerch et al., 2020). Animals can sometimes become an extension of the victim, such that hurting the animal in turn hurts the victim (Fitzgerald et al., 2019). Abusers can thus use CAs as a way to exert power and control over their victims. For instance, the abuser can coerce the victim into staying (or returning) with threats of harm to the animal (Castillo, 2013; Giesbrecht, 2022a, 2022b). Thus, the actual or threatened abuse toward the CA can be a component of relationship abuse.

## CA Programs

As a result of the well-established link between IPV and animal maltreatment, many domestic violence agencies have developed policies, programs, and/or arrangements to address the issue so that human and nonhuman victims are able to flee abusive relationships safely. Some agencies allow CAs to stay with the survivor in the survivor’s room or in another on-site location (e.g., pet area, kennel on the property). Other agencies have established relationships with off-site organizations or providers who offer refuge for the CAs (e.g., veterinarians, Humane Society, foster programs). However, establishing a CA program is the exception rather than the rule. Based on a survey given to domestic violence shelter staff in Canada, Stevenson et al. (2018) found that over 75% of staff were aware of women in the community who had refused to come to the shelter because they could not bring their CAs. In this same study, 72% of shelter staff indicated that their organization had no questions about animals on their intake forms, and only 33.6% of staff stated that the shelter they worked at had an official CA policy (Stevenson et al., 2018).

A lack of CA programs within shelters was also confirmed by the results of a study conducted by Gray et al. (2019). The authors analyzed 337 public websites of domestic violence shelters in Canada to see what the shelters were communicating about IPV and animal abuse as well as what services they offered in terms of CA programs. Only 1% of shelter webpages reported offering on-site CA programs, and less than half of the websites mentioned CAs at all (Gray et al., 2019). The failure to communicate and/or advertise CA programs on the phone and on websites may be a deterrent for women seeking shelter with their CAs.

## Identifying Barriers to Implementing CA Programs

Although there has been an increase in the number of domestic violence shelters that have established CA programs, a number of barriers to implementing CA programs have also been identified. Krienert et al. (2012) conducted a study most similar to the present study. In their study, 767 domestic violence shelters participated in a nationwide e-survey to assess the services available to women fleeing from domestic abuse with their CAs. Results showed that for shelters that did not have any sort of CA services, the most common reasons for this were lack of resources (50%), lack of space (40%), safety issues (27%), medical concerns (24%), and lack of awareness (5%; Krienert et al., 2012). Additionally, only 6% of shelters reported having on-site CA programs, and these programs still had restrictions such as maximum lengths of stay, CA size restrictions, and limits on the number of CAs that can accompany residents (Krienert et al., 2012). However, given that section 12502 Protecting Animals with Shelter (PAWS) of the Agriculture Improvement Act, which created a grant program to help domestic violence shelters better assist the needs of survivors with CAs, was signed into law in 2018 in the United States (Pub. L. No. 115-334) and nonprofit organizations such as RedRover began offering grants to shelters to establish CA programs, it is important to examine whether the barriers Krienert et al. (2012) identified still apply. Identification of currently existing barriers to implementing CA programs is needed to address such barriers and to increase the number of CA programs offered by domestic violence shelters.

In addition to Krienert et al.’s (2012) nationwide investigation of CA programs in the United States, a handful of other regional evaluations have been conducted. For instance, Komorosky et al. (2015) conducted a mixed-methods study using both surveys and in-depth interviews to examine CA programs and policies in domestic violence shelters and transitional houses in California, USA. Of 57 survey responses analyzed, none of the respondents reported regularly allowing CAs to stay with their guardians, except for special circumstances in which service animals were allowed or if special consideration was given to a client. The authors found that concerns related to CA safety, placement challenges (e.g., difficulty arranging for CAs to be sent to foster placement or kennels), and cost/resources were the most prevalent barriers to implementing CA programs (Komorosky et al., 2015).

Similar barriers have been identified in other studies, including in a sample of interpersonal violence advocates and administrators in the Western United States (*N* = 21; Hageman et al., 2018); Society for the Prevention of Cruelty to Animals (SPCA) staff in New Zealand (*n* = 4; Gillespie-Gray, 2017); mixed samples of animal welfare providers (*n* = 43 and *n* = 32, respectively, from SPCAs, humane societies, etc.) and IPV service providers (*n* = 128 and *n* = 51, respectively, from shelters/transition houses, victim services, etc.) recruited in urban, rural, and northern communities in Saskatchewan, Canada (Giesbrecht, 2022a, 2022b, Giesbrecht et al., 2025; Wuerch et al., 2021); and emergency shelter staff (*N* = 20) from Ontario, Canada (Matsuoka & Sorenson, 2022). Specifically, the following barriers were identified: safety and/or health concerns, including risk of perpetrators harming those who attempt to provide care for the animals (Giesbrecht, 2022a; Hageman et al., 2018; Matsuoka & Sorenson, 2022; Wuerch et al., 2021); space concerns (e.g., lease or shelter restrictions, space restrictions, difficulty housing larger animals; Giesbrecht, 2022a, 2022b; Gillespie-Gray, 2017; Hageman et al., 2018; Matsuoka & Sorenson, 2022; Wuerch et al., 2021); limited resources (e.g., limited finances and human resources; Giesbrecht, 2022a; Gillespie-Gray, 2017; Hageman et al., 2018; Matsuoka & Sorenson, 2022; Wuerch et al., 2021); legal concerns (Giesbrecht, 2022a; Wuerch et al., 2021); lack of transportation for animals from rural areas (Giesbrecht, 2022a, 2022b; Wuerch et al., 2021); lack of training in caring for animals (Giesbrecht, 2022a, 2022b); and increased workload (Giesbrecht, 2022a; Matsuoka & Sorenson, 2022). Respondents in studies by Giesbrecht (2022a), Giesbrecht et al. (2025), and Wuerch et al. (2021) also offered some suggestions to overcome these barriers, such as increased funding; providing more options for animal safekeeping, including accommodating animals in domestic violence shelters and having more short-notice foster homes; strengthening existing and building new partnerships between IPV and animal welfare agencies; improving safety procedures; issuing more emergency intervention orders for those with CAs (which are temporary orders that allow victims to stay in the home while the perpetrator is removed from the residence); and effective information sharing and “cross-reporting between child protection and animal protection agencies” (Giesbrecht, 2022a, p. NP16951). It was also found that shelters in rural and remote areas may face more challenges than those in urban areas (Giesbrecht, 2022a, 2022b; Giesbrecht et al., 2025; Wuerch et al., 2021). It is notable that much of the research on barriers to developing CA programs within domestic violence shelters has been conducted in Canada, with a few recent studies having been conducted in Saskatchewan. There is thus a need to further investigate the status of such barriers in the United States, especially given the recent enactment of the PAWS section of the Agriculture Improvement Act (2018), so that funding offered through PAWS can meaningfully address existing barriers.

## Current Study

Drawing upon past research, we thus aimed to address two research questions using data from phone or online surveys completed by over 400 shelters across the United States that did not have any on-site CA programs at the time of the assessment: (1) What are the barriers to implementing on-site CA programs in domestic violence shelters in the United States? and (2) Do the barriers and the number of barriers endorsed differ across (a) rural versus urban locations and (b) geographical regions of the United States? We also explored whether (c) the number of barriers endorsed differed in rural versus urban locations within each of the four U.S. geographical regions. Given that certain barriers may be more common in certain settings, as shown by Giesbrecht (2022a, 2022b) and Giesbrecht et al. (2025), it is important to assess for these potential patterns. Identifying potential region-specific barriers could inform the development of strategies for mitigating barriers that restrict domestic violence shelters from offering on-site CA programs.

## Methods

### Participants

After receiving ethics clearance, a list of 1,765 U.S. domestic violence shelters was compiled by visiting each state’s and 3 U.S. territories’ (i.e., Guam, Puerto Rico, Virgin Islands) domestic violence coalition websites as well as www.domesticshelters.org and www.womenslaw.org. Because no agencies within a U.S. territory participated in the study, we refer only to the United States hereafter. Of this initial list, a team of trained undergraduate (*n* = 6) and graduate (*n* = 3) research assistants contacted a total of 1,765 shelters. Reasons for noncompletion included that interviewers were not always able to speak to a person at the agency (*n* = 440), agency staff did not always follow up with interviewers after indicating they would call us back (*n* = 257), some agencies declined to complete the interview (*n* = 51), some phone numbers were for multiple agencies (*n* = 166), and some agencies did not provide enough usable data or other issues arose with the data (*n* = 148). Given the present study’s focus on barriers to developing and/or implementing CA programs, only agencies that did not currently have on-site CA programs (*n* = 532; 75.8%) were included in analyses. Moreover, agencies that had missing data (*n* = 31) or that provided the following responses when asked to identify the barriers they perceived to be interfering with the implementation of CA programs in their current agencies were removed from analyses (*n* = 96): *unsure*, *not applicable*, *pets not a problem*, and *no barriers*. Thus, the final sample consisted of 405 agencies that identified barriers to implementing CA programs. Agencies from all 50 states completed surveys, and the number of agencies per state ranged from one agency (Hawaii and Utah) to 26 agencies (Pennsylvania). However, no agencies in Delaware or Nevada identified any barriers to implementing CA programs; Delaware and Nevada were thus the only states not represented in the current study.

Of the 405 agencies, 339 (83.7%) completed the interview by telephone and 66 (16.3%) answered the questions online. The majority of agencies were domestic violence emergency shelters (*n* = 388) followed by domestic violence nonhousing direct services (*n* = 286), domestic violence housing services (*n* = 84), and domestic violence legal services (*n* = 11). It should be noted that many agencies provided multiple services. Anyone who answered the phone and was willing to participate was welcome to do so. Participants who answered the questions via telephone or online had a number of different titles within the organizations/shelters for which they were interviewed; in fact, there were so many titles that the majority were coded as *Other* (212; 52.3%), followed by program director or administrator (*n* = 67; 16.5%) and executive director (*n* = 46; 11.4%) to name a few. No compensation was offered for participation.

### Procedure

Trained undergraduate and graduate researcher assistants called the numbers on the list and used a prescribed semi-structured script to interview the organizations over the phone upon contact. Data were collected in June through September 2019. Informed consent was obtained orally, and participants were reminded of their right to discontinue participation or to refuse to answer any questions. The interviewers asked the questions from the script and recorded the responses into an online Qualtrics survey. In the case that an organization could not be reached by phone, but an email address was available, researchers emailed the open-ended interview questions to respondents to be completed and emailed back to the researchers. During the interviews, participants were also allowed to elect to complete the interview questions online rather than over the phone.

### Measures

Research assistants were trained by a member of the research team on telephone interview protocols and use of the database interviewers entered respondents’ data into (e.g., Qualtrics) through participation in role plays, data coding and recording exercises, and simulated interviews before completing interviews with participants. They used a prescribed script while conducting the brief semi-structured interviews over the phone. The interview consisted of 21 questions, but the number of questions per interview varied due to participants’ responses to various questions and question branching. For example, if an organization did not have an on-site CA program, they were not asked any further questions pertaining to on-site CA programs. On average, interviews took about 10 to 15 min to complete. In the present study, besides demographic questions, we focused on three of the interview questions.

#### Barriers to CA Programs

For agencies that indicated they did not have any CA programs, respondents were asked, Are there any specific barriers, considerations, or limitations that contributed to your agency’s decision to not offer on-site pet services? Twelve content codes were created, including (a) no; (b) yes, location barriers (we are an urban organization); (c) yes, location barriers (we are a suburban organization); (d) yes, location barriers (we are a rural organization); (e) yes, landlord issues (our building owner does not allow animals); (f) yes, zoning issues; (g) yes, insurance issues; (h) yes, space issues; (i) yes, financial issues; (j) yes, safety issues; (k) other (specify); (l) do not know. Other responses were either coded into existing categories or new categories. As indicated above, participants who provided no and do not know responses were removed from analyses.

#### Rural Versus Urban Location

In order to analyze the main barriers by rural versus urban location, the cities of each agency were coded as rural or urban depending on the population size. Cities with a population less than or equal to 2,500 were coded as rural and cities of more than 2,500 residents were coded as urban, as per the classifications indicated on the U.S. Census website (United States Census Bureau, 2020).

#### Region of the United States

To assess whether agencies located in different regions of the United States endorsed different barriers to implementing CA programs, states were grouped into the four U.S. Census Bureau regions: Northeast, Midwest, South, and West (United States Census Bureau, 2021).

### Data Analysis

Open-ended interviews were analyzed using directed content analysis to code the data into pre-determined anticipated codes. For responses that did not map onto the pre-determined codes, new codes were created. To identify potential barriers to implementing on-site CA programs in U.S. shelters, data were analyzed primarily using descriptive statistics (e.g., frequencies/percentages). We used chi-square tests to assess whether certain barriers applied more to rural versus urban locations and to certain geographical regions of the United States and *t* tests and one-way analyses of variance to examine whether the number of barriers endorsed differed as a function of location, geographic region, and rural versus urban location within geographic region. We conducted analyses with IBM SPSS Statistics (Version 26) predictive analytic software (IBM Corp., 2019).

## Results

To address the first research question, *What are the barriers to implementing on-site CA programs in domestic violence shelters in the United States*, we calculated descriptive statistics to find (a) the prevalence of barriers overall; (b) the number of agencies that endorsed multiple barriers; and (c) the mean number of barriers endorsed.

### Prevalence of Barriers

A total of 29 specific barriers were endorsed by the agencies, and these barriers were then grouped into 5 major categories: health and safety, space, resources, policies, and workplace issues, with the 3 categories of health and safety (12 barriers), space (8 barriers), and resources (4 barriers) encompassing the greatest number of different barriers (see Table 1). Similarly, the categories most frequently endorsed overall were health and safety (*n* = 376, 43.6%), space (*n* = 350, 40.6%), and resources (*n* = 113, 13.1%; Table 1). It is noteworthy that there was near equal endorsement for health and safety and space. The modal responses for barriers within the health and safety category were safety issues (44.4%), allergies (24.5%), and insurance liability issues (15.4%). Within the space category, the most endorsed barriers were space issues (76.0%), location within a rural setting (6.0%), and logistics associated with space (e.g., yard size, age of building; 4.3%). For the resources category, modal responses included financial issues (74.3%) and insufficient staff (15.0%). Frequently endorsed responses within the policies category included an agency’s requirement for CAs to be service animals (53.3%) and other agency policies related to CAs or the lack thereof (33.3%). Lastly, responses coded as workplace issues included concerns about destruction of property (e.g., furniture) or noise (55.6%) and staff and/or resident preferences (44.4%; see Table 1). Thus, overall, a wide range of barriers were endorsed. Having said that, as indicated above, although they were not included in the current analyses, it is noteworthy that a small proportion of the agencies that did not have on-site CA programs in place (*n* = 22) also reported that they saw no notable barriers to implementing on-site CA programs.

**Table 1.** Frequencies of Barriers to Implementing On-Site Companion Animal Programs.

Barriers	*N*	Percentage of Barriers Within Subcategory	Percentage of Total Barriers
Health and safety	376		43.6
Safety issues	167	44.4	19.4
Allergies (other residents and/or staff)	92	24.5	10.7
Insurance issues (including liability concerns)	58	15.4	6.7
Children present	17	4.5	2.0
Resident fears of pets (including children)	9	2.4	1.0
Health concerns of residents (other than allergies)	7	1.9	0.8
Hygiene/cleanliness concerns	6	1.6	0.7
Environment not safe/good for pets	6	1.6	0.7
Behavior issues of pets (including risk of; how pets will react)	4	1.1	0.5
Previous bad experience with pets on-site	4	1.1	0.5
Concerns about pet health (e.g., lack vaccines)	4	1.1	0.5
Pests (e.g., fleas)	2	0.5	0.2
Space	350		40.6
Space issues	266	76.0	30.8
Location (rural)	21	6.0	2.4
Logistics of space (e.g., set-up, yard size, age of building)	15	4.3	1.7
Location (urban)	14	4.0	1.6
Communal/shared space (including some bedrooms)	14	4.0	1.6
Landlord does not allow animals	14	4.0	1.6
Zoning issues	3	0.9	0.3
Location (suburban)	3	0.9	0.3
Resources	113		13.1
Financial issues	84	74.3	9.7
Insufficient staff (including inadequate training)	17	15.0	2.0
Government or funders policies	9	8.0	1.0
Insufficient resources to help residents care for pets	3	2.7	0.3
Policies	15		1.7
Pets must be service animals	8	53.3	0.9
Organization policies (or lack thereof)	5	33.3	0.6
Concerns about pet definitions (e.g., “fake” service animals)	2	13.3	0.2
Workplace issues	9		1.0
Destructive (e.g., furniture) and noise concerns	5	55.6	0.6
Preference of staff/residents (including being bothered)	4	44.4	0.4
Total	863		

*Note*. Because some agencies endorsed multiple barriers, percentages summed across categories exceed 100%.

When considering multiple barriers to implementing CA programs, almost one half of the agencies (*n* = 258, 45.5%) endorsed more than one specific barrier. Of the number of barriers endorsed, agencies reported from one to eight specific barriers (*M* = 2.13, *SD* = 1.17). Although identifying one barrier was the most common number of barriers endorsed (*n* = 147, 36.3%), about one fourth of agencies endorsed two specific barriers (*n* = 131, 24.6%), which was the median number of barriers endorsed.

### Main Barriers by Rural Versus Urban Location

Consistent with the overall findings, examination of barriers by rural versus urban locations (Research Question 2a) suggested that, in both rural and urban locations, over one half to three fourths of agencies endorsed space and health and safety concerns as the main barriers to implementing CA programs. Concerns related to resources were also frequently endorsed in agencies situated in both locations. Table 2 shows the frequencies of all main barrier categories endorsed across rural versus urban locations as well as results of chi-square tests that assessed differences across location types. The cell sizes were too small to adequately assess differences by location for the policies category. However, of the remaining barriers, none was significantly more likely to be endorsed by agencies in rural versus urban locations. Thus, the type of barriers endorsed did not differ across agency location.

**Table 2.** Frequencies of Barriers by Rural Versus Urban Location and Analysis of the Number of Barriers Endorsed Across Rural Versus Urban Location.

Barriers	Rural (*n* = 27)		Urban (*n* = 378)		*df*	χ^2^	*p*
	*n*	%	*n*	%			
Space	20	6.6	282	93.4	1	.004	.95
Health and safety	16	6.7	223	93.3	1	.001	.98
Resources	5	4.8	99	95.2	1	.777	.38
Workplace issues	2	22.2	7	77.8	1	2.344^a^	.13
Policies	0	0	13	100.0	—	—	—

*Note*. Because some agencies endorsed multiple barriers, percentages summed across categories exceed 100%.

a Fisher exact tests were reported for the Workplace Issues category due to insufficient cell sizes for some of the cells. A chi-square test could not be calculated for policies due to insufficient cell count in one of the cells.

### Main Barriers by Region of the United States

Table 3 shows the frequencies of main barrier groups across geographical regions (Research Question 2b). Issues related to space were endorsed most frequently by agencies located in all four regions of the United States. In fact, 71% to 83% of agencies in regions across the United States reported space as an issue of concern. Agency staff in the Northeast rated concerns about space most frequently. Similarly, barriers related to health and safety concerns were raised by over half of the agencies in each of the four regions of the country, with almost equal endorsement across the regions. Resources were the third most prevalent barrier, raised by roughly 22% to 30% of agencies in each of the four regions. Only a few agencies cited issues related to workplace issues (1.3%–4.0%) or agency policies (1.3%–6.1%) as barriers.

**Table 3.** Number of Domestic Violence Shelters That Endorsed Barriers to Implementing On-Site Companion Animal Programs Across Regions of the United States.

Barriers	Total *n*s (*N* = 405)	Northeast (*n* = 75; 18.5%)		Midwest (*n* = 140; 34.6%)		South (*n* = 120; 29.6%)		West (*n* = 70; 17.3%)		χ²	*p*
		*n*	%	*n*	%	*n*	%	*n*	%		
Space	302	62	20.5	103	34.1	85	28.1	52	17.2	3.55	.31
Health and safety	239	45	18.8	83	34.7	73	30.5	38	15.9	.85	.84
Resources	104	21	20.2	31	29.8	36	34.6	16	15.4	2.60	.46
Workplace issues	9	1	11.1	6	66.7	2	22.2	0	0.0	3.66^a^	.26
Policies	13	1	7.7	2	15.4	5	38.5	5	38.5	5.47^a^	.11

*Note.* Because some agencies endorsed multiple barriers, percentages summed across categories exceed 100%.

a Fisher exact tests were reported for the Workplace Issues and Policies categories due to insufficient cell sizes for some of the cells. The degrees of freedom for all categories were 3.

### Differences in Barriers Across U.S. Regions

To further assess Research Question 2b, we conducted chi-square analyses to determine whether endorsement of barriers differed across the U.S. geographical regions. Some of the cell sizes were once again too small to adequately assess differences by geographical region for the policies category as well as for the workplace issues category. However, when considering other barriers, the types of barriers endorsed did not differ across geographical regions (Table 3). Thus, similar barriers to implementing CA programs within domestic violence shelters were reported across different regions of the country.

Finally, we conducted a one-way analysis of variance to determine whether the total number of barriers endorsed differed by U.S. region (Research Question 2b) and a series of *t*-test analyses to assess whether the number of barriers endorsed differed by rural versus urban locations within each of the four U.S. regions (Research Question 2c). The results showed that there were no significant differences in the total number of barriers endorsed across U.S. regions, *F*(3, 1) = 0.15, *p* = .93, η^2^ = .001, nor across rural versus urban locations within each of the geographical regions (see Table 4). Thus, taken together, agencies serving individuals fleeing from abusive relationships located across the United States reported similar concerns and a similar number of concerns about the development and implementation of on-site CA programs in their agencies.

**Table 4.** Number of Barriers Endorsed for Rural Versus Urban Domestic Violence Shelters Within Regions of the United States.

Region	Rural			Urban			*t*	*df*	*p*	Hedge’s *g*
	*n*	*M*	*SD*	*n*	*M*	*SD*				
Northeast	6	1.83	0.98	69	2.23	0.99	−0.95	73	.35	−0.40
Midwest	13	2.62	1.39	127	2.05	1.21	1.59	138	.12	0.46
South	6	1.50	0.55	115	2.14	1.14	−1.37	118	.17	−0.57
West	2	2.50	0.71	68	2.15	1.31	0.38	68	.71	0.27

## Discussion

In the current study, we examined the prevalence of specific barriers to implementing on-site CA programs in U.S. domestic violence shelters. Consistent with past research conducted across multiple nations (i.e., Canada, New Zealand, the United States), numerous barriers were endorsed (Giesbrecht, 2022a, 2022b; Gillespie-Gray, 2017; Hageman et al., 2018; Komorosky et al., 2015; Krienert et al., 2012; Wuerch et al., 2021). In fact, almost 30 separate barriers were identified, which collapsed into 5 main categories. The nonsignificant differences in endorsement of barriers by rural versus urban location, geographic region, and rural versus urban location within U.S. geographic region suggest that domestic violence shelter staff across all areas of the United States report similar types of barriers. Although the initial goal of the current study was to gather information on potential location-specific barriers in order to offer recommendations for addressing the most prevalent barriers in each area, the present findings indicated that there may actually be a consistent set of barriers, regardless of city size and region. These findings thus suggest that assistance in developing and implementing on-site CA programs may not need to be tailored to specific areas and regions but may be implemented at a national level.

The most prevalent barriers found in the present study across rural and urban locations as well as across all four geographical regions of the United States were health and safety, space, and resources. Barriers related to workplace issues and agency-based policies were less common, but there were still a number of agencies that reported these barriers as well. These results are consistent with the findings of studies conducted in the United States (Hageman et al., 2018; Krienert et al., 2012), Canada (Giesbrecht, 2022a, 2022b; Stevenson et al., 2018; Wuerch et al., 2021), and New Zealand (Gillespie-Gray, 2017). In fact, the three most common barriers endorsed by participants in Krienert et al.’s (2012) sample were also space, health and safety, and resources. Interestingly, Krienert et al. (2012), who conducted their investigation prior to the introduction of RedRover’s Safe Housing grants and the PAWS section of the Agriculture Improvement Act (2018), reported that concerns related to resources were the most prevalent barrier. Thus, the prevalence of the barriers may have changed over time, and such changes may have been influenced by funding provided by various organizations (e.g., RedRover, PetSmart Charities) and new legislation.

It is noteworthy, that overall and across types of locations and regions of the United States, health and safety and space concerns were endorsed as the most common barriers. Moreover, although health and safety concerns were reported slightly more frequently overall, safety concerns were the most frequently endorsed concerns when considered as a function of location and region of the United States. Issues raised by agency staff related to both health and safety and space included a range of concerns for each category. For instance, 12 specific health and safety concerns were raised, including risks associated with the CAs harming clients, children, or staff; allergies; liability issues and/or litigation that may result from harm caused by the CAs; and other health problems resulting from exposure to the CAs. Similarly, in terms of space, staff mentioned constraints produced by type of location (e.g., rural vs. urban); size of buildings, rooms, and land plots; limitations of communal space; zoning ordinances; and landlord restrictions. Thus, the issues of health and safety and space appear to be multifaced, indicating that multiple solutions may need to be implemented to overcome these barriers. Increased funding may be particularly helpful as it could help address many of the issues.

Another important finding from the present research is that there were some agencies that did not feel there were any notable barriers to developing and/or implementing CA programs within their agencies (reflected by their responses of “no barriers,” “not a problem,” etc.), even though they did not currently have an existing CA program in place. This is promising information and suggests that because these agencies did not perceive there to be any barriers, they may be open to developing/implementing CA programs. Similarly, the modal number of barriers endorsed by agencies was one barrier. This suggests that the obstacles to developing and implementing pet programs may not be too great for some agencies and that if a solution for dealing with the one barrier could be implemented, agencies may be able to serve survivor victims who are fleeing with their CAs.

The present research also showed that, for some agencies, there were multiple barriers preventing them from being able to accommodate survivors with CAs. Some agencies, albeit a minority, endorsed up to eight separate barriers. The wide range in number of barriers shows that it may be less possible for some agencies than others to establish CA programs. For instance, as suggested by Wuerch et al.’s (2021) research, agencies with limited staff, space, and resources servicing clients who have numerous livestock animals as well as several CAs may not be able to develop programs that can offer safety to all human and nonhuman animals in abusive relationships within their communities. Thus, alternate models of care and safety may need to be established to address these complex scenarios.

Lastly, given that resources were the most frequently endorsed barrier in Krienert et al.’s (2012) study (by 50% of agencies surveyed in California) versus the third most frequently endorsed barrier in the current study (by 13% of agencies surveyed across the United States), there may be some preliminary evidence showing that attention brought to the need for CA programs in domestic violence shelters by nonprofit organizations (e.g., RedRover, PetSmart Charities) and the Agriculture Improvement Act (2018) helped addressed domestic violence shelters’ concerns about having adequate resources to implement CA programs. This finding is encouraging and provides support for the continuation of relevant legislation and funding programs.

### Limitations

The main limitation of the present study was the moderately low response rate. Of 1,740 agencies, only 702 responded, yielding a response rate of 40.3%. Due to the lack of responses from agencies that declined to participate or that did not finish the study, our results may be biased towards agencies that see CA concerns as important enough to warrant participation. In addition, it is also possible that nonparticipating agencies may have been particularly under-resourced, precluding them from being able to take part in the study. Another limitation was the small number of participating agencies located in rural areas. Of the 405 agencies that endorsed barriers, only 30 (6.9%) were located in rural areas. This may limit the generalizability of the findings to agencies in rural areas. Although rural areas are less likely to have one or more domestic violence shelters simply due to lower population density, the present results may be more reflective of domestic violence service agencies in urban and/or urban cluster (population of 2,500–50,000 people) areas. Last, although we chose to use the U.S. Census data definition for delineating rural versus urban locations, our results may have differed had we used an alternative operationalization (e.g., the Economic Research Service of the United States Department of Agriculture uses the term rural to refer to nonmetropolitan areas; United States Department of Agriculture, n.d.).

### Contributions to Research on the Link Between IPV and Animal Maltreatment and Barriers to On-Site CA Programs

Despite these limitations, the current study made several contributions to the literature. First, it provided the first known nationwide scan of the barriers U.S. domestic violence shelters face in developing on-site CA programs. Second, because of the systematic approach we took to locating and contacting all publicly identified shelters, our data were inclusive not only of shelters located in all regions of the United States but of shelters of different sizes, formats, and types (e.g., those established for survivors living on reservations in North Dakota, for Spanish-speaking survivors in New Mexico). Third, we provided the first analysis of whether barriers differ in communities of varied sizes or in different regions of the United States.

### Future Research

Several lines of research are needed to expand upon the current findings. Given the smaller proportion of shelters that were based in rural versus urban communities in the current sample and research that has suggested that shelters in rural and remote locations may experience greater and/or unique barriers to developing CA programs (Giesbrecht, 2022a, 2022b; Giesbrecht et al., 2025; Wuerch et al., 2021), additional research is needed to verify whether shelters in these regions do or do not experience more and/or distinct barriers. Further research focused on the differing types of agencies (e.g., emergency shelter, transitional housing) may also be beneficial in determining possible next steps toward implementing pet programs. In relation to type of agencies, further research may also focus on the differences in barriers within different types of existing pet programs (e.g., on-site, off-site). Finally, research is needed to explore what makes successfully implemented pet programs effective. It is thus important for shelters to conduct program evaluations of their CA programs, for funding agencies to encourage and support program evaluation, and for shelters to share what is learned from these evaluations with other shelters, community partners, funding agencies, and academic researchers. Such research may provide information as to the process by which agencies were able to overcome various barriers, and, in turn, possible solutions to reducing or eliminating the barriers discussed in the literature.

### Implication for Practice

Overall, findings from the current study suggest that many shelters could develop and implement a CA program within their organizations with relative ease. There are now manuals that provide specific instruction and recommendations for selecting the most appropriate CA program for organizations (e.g., based on space, location, staffing, residents’ needs); establishing buy-in and participation by staff, residents, and community partners; estimating costs and securing funding and donations; designing the program and necessary policies and procedures (e.g., including those that minimize risks and potential harms, promote health, address liability issues, establish expectations with staff, residents, and community partners); and implementing the program. These include Allie Phillips’ *Sheltering Animals and Families Together (SAF-T) Start-up Manual, Third Edition* (2020); the Pet Friendly *Shelter Viability Report* (Praxis Consulting Ltd., 2021), which was jointly sponsored by the Saskatchewan SPCA, PATHS (Provincial Association of Transition Houses and Services of Saskatchewan), and STOPS to Violence (Saskatchewan Towards Offering Partnership Solutions to Violence); and a toolkit on sheltering pets and people created by the British Columbia Society of Transition Houses (2022). These resources, in addition to funding for CA programs, effective coordination among multisectoral teams (e.g., shelter staff, animal welfare organizations, law enforcement, veterinary care staff, health and mental health service providers, transportation services, financial institutions), supportive legislation, and increased advocacy for and education about the link between IPV and CA maltreatment, are imperative for ensuring safety to both human and animal IPV victims (Giesbrecht, 2022c).

### Conclusion

Greater knowledge and awareness of the link between IPV and animal maltreatment has made the need for CA programs in domestic violence shelters direly apparent. Findings from the current study suggest that novel solutions are needed to address concerns primarily related to space, health and safety, and financial and other resources when developing on-site CA programs. The present research also suggests that solutions targeted at specific types of communities may not be needed, but that more general strategies may be appropriate. Lastly, there is some evidence to suggest that funding and legislation focused on these efforts may prove beneficial in providing IPV survivors and their CAs with the safety they need and deserve.
